# Post esophagectomy diaphragmatic hernia: a case report of a rare cause of acute respiratory distress

**DOI:** 10.1186/s13019-018-0802-x

**Published:** 2018-11-15

**Authors:** Valérie Lamontagne, Valérie Lafrenière-Bessi, Arthur Vieira, Éric Charbonneau, Paula A. Ugalde, Frédéric Jacques

**Affiliations:** 1 Service of Cardiac Surgery, Multidisciplinary Department of Cardiology, Institut universitaire de cardiologie et de pneumologie de Québec, Université Laval, QC, Canada; 2 Service of Thoracic Surgery, Multidisciplinary Department of Respirology, Institut universitaire de cardiologie et de pneumologie de Québec, Université Laval, Quebec City, Canada

**Keywords:** Diaphragmatic hernia, Respiratory distress, Post-esophagectomy, Cardiac surgery

## Abstract

**Background:**

Diaphragmatic hernia is frequent among the elderly and is usually associated with mild chronic digestive and respiratory symptoms.

**Case presentation:**

An elderly post-esophagectomy male patient, in the early postoperative period of cardiac surgery, presented with acute respiratory distress. An emergent surgery was performed to reduce a giant diaphragmatic herniation.

**Conclusions:**

An acute transhiatal herniation can cause serious respiratory impairment; surgical repair should be considered in select patients of cardiac surgery.

## Background

Diaphragmatic hernia is a benign condition that manifests with chronic dyspnea and intermittent gastrointestinal symptoms, such as epigastric pain, postprandial fullness and nausea [1, 2]. Acute respiratory distress associated with a giant diaphragmatic herniation of abdominal contents is unusual in the early postoperative period of cardiac surgery. Atypical symptoms, detailed clinical management and the surgical hernia repair in an elderly post-esophagectomy male patient are described.

## Case presentation

A 74-year-old male, who had undergone 3 stent coronary implantation procedures in the previous 6 months, presented to the hospital with progressive dyspnea and recurrent chest pain. The patient’s medical history was noted for esophageal cancer treatment that consisted of a radical esophagectomy, gastric pull-up followed by chemotherapy and radiotherapy. Esophageal cancer recurrence was ruled out. A transthoracic echocardiography revealed severe aortic regurgitation, moderate mitral regurgitation and a left ventricular ejection fraction of 44%.

**Fig. 1:**
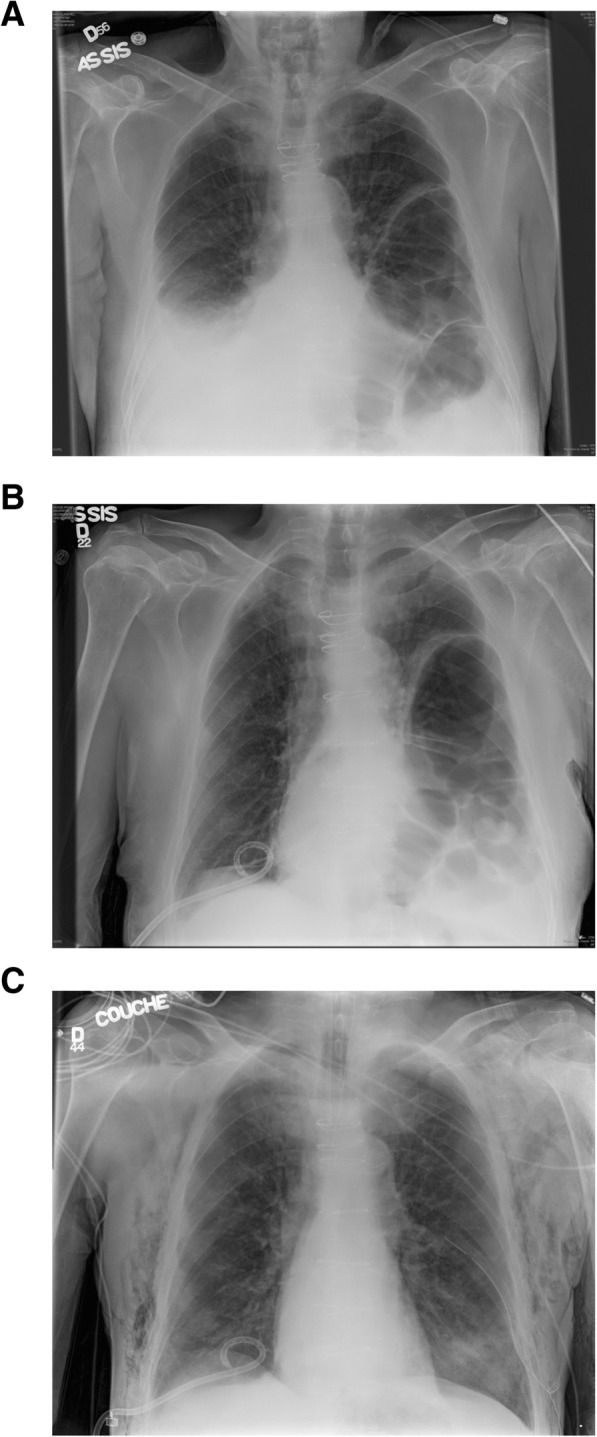
Anterior to posterior chest roentgenogram showing: (**a**) recurrent right chylothorax combined with the left intrathoracic herniated colon (upright sitting position); (**b**) right pigtail catheter insertion and chylothorax drainage (upright sitting position); (**c**) resolution of both the chylothorax and the herniated colon (supine position)

**Fig. 2:**
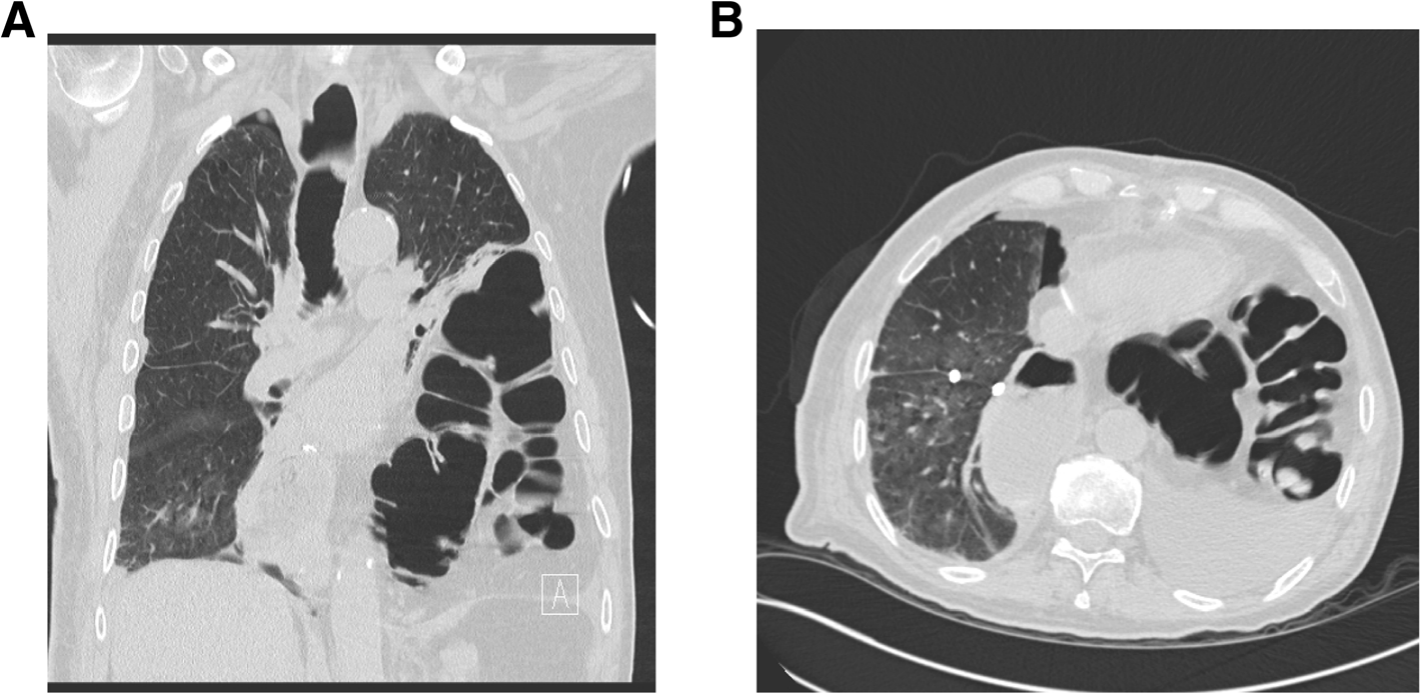
Chest computed tomography depicting left lung atelectasia as a consequence of colic herniation in the chest: **a** coronal view); (**b**) Transverse view

The patient underwent a dual valve replacement procedure with a bioprothesis aortic valve (23 mm Magna Ease, Edwards Lifesciences, CA, USA) and mechanical mitral valve (25 mm ON-X, CryoLife, GA, USA). The patient required 7 days of inotropes and intensive unit care. On postoperative (PO) day 8, a right-sided chylothorax was diagnosed, and treated with simple drainage and low-fat medium chain triglycerides diet. On PO day 18, the patient evolved with acute respiratory deterioration and hypoxemia. Chest auscultation revealed peristaltic sounds on the left side. Chest x-ray revealed right pleural effusion and abdominal contents within the left chest cavity (Fig. 1a). Despite pleural effusion drainage, the patient had only slightly improved the respiratory status (Fig. 1b). A chest computerized tomography confirmed the presence of a large portion of the transverse and descending colon in the left hemithorax with no radiological sign of intestinal necrosis (Fig. 2). The diaphragmatic hernia measured 15 cm and filled the whole transverse dimension of the left chest on the anterior-posterior view. A transthoracic echocardiogram ruled out acute cardiac complications. Clinical deterioration was evidenced by increased oxygen requirements to 5 L/min, tachypnea, tachycardia and confusion.

Urgent diaphragmatic hernia repair was indicated and performed by laparoscopy. The patient was placed in a dorsal position with hyperextension of the upper third of his abdomen. Laparoscopic surgery was performed through two 12 mm trocars on the left and right paraumbilical region and three 5 mm trocars were used in the subcostal region, one on the right side and two on the left. The 10 mm 30G camera was inserted through the left paraumbilical incision. A large quantity of peritoneal adherences was taken down with harmonic synergy blades (Ethicon, OH, USA) under direct vision. A large diaphragmatic hernia was identified with a large portion of the transverse colon and omentum within the left chest cavity. Once the majority of the colon was reduced the dissection of the hernial sac from the right and from the left hiatal pillars toward the mediastinum and the apex of the left chest. Pealing the sac off was mandatory for a complete reduction and repair of the hernia. As expected, the apical portion was the most laborious however with proper exposure the dissection was safely performed. The colon stayed passively in the abdominal cavity. The repair of the hiatal hernia was performed by approximating the left and right pillars with non-absorbable stiches. A Biodesign Hiatal Hernia Graft (Cook Medical, IN, USA) was placed surrounding the hiatus. A prolene mesh (Ethicon, OH, USA) was used to close the anterior space. Both grafts were fixed with tacker fixation device (Medtronic, MN, USA). The final result was satisfactory.

The patient had postoperative ischemic colitis and interstitial alveolar left lower lobe infiltrate. This was managed with a conservative treatment based on antibiotics and parenteral nutrition. A Pleur-X chronically indwelling catheter system (Becton Dickinson, NJ, USA) was installed in the patient before discharge, 3 months after surgery. One year after discharge, the patient was readmitted with increased dyspnea. A right sided chylothorax, secondary to Pleur-X infection, was diagnosed. The drainage system was changed, antibiotic treatment was given for 2 weeks and the patient is now doing well.

## Discussion and conclusions

Though the incidence of post-esophagectomy hiatal hernia is increasing, the occurrence of acute giant hiatal hernia complicated with acute respiratory distress is rare [3, 4]. Asymptomatic patients with post-esophagectomy hernias are usually observed [5]. In cases of respiratory symptoms, related to aspiration with gastroesophageal reflux disease or shortness of breath caused by mechanical pressure induced by intrathoracic herniation, surgical treatment is indicated [6, 7].

In this case, an urgent surgical repair was needed to treat respiratory distress caused by an acute hiatal hernia in the early postoperative period of cardiac surgery. The common causes of respiratory distress, such as cardiac tamponade, pleural effusion, fluid overload and prosthesis thrombosis or dehiscence, were excluded. A chest X-ray followed by a computerized tomography scan confirmed a herniated colon into the left chest cavity with complete lung atelectasis.

Surgical treatment of diaphragmatic hernia is usually performed after recurrent and dramatic symptoms [1, 8]. Physicians should keep in mind that a large hiatal hernia can develop in post-esophagectomy patients and cause atypical symptoms such as chronic dyspnea, exercise intolerance and recurrent chest pain. Rapid respiratory decompensation in patients with indolent diaphragmatic hernia can occur with changes in the respiratory physiology, especially with acute changes in intrathoracic pressure. Risk assessment for surgical repair of giant hiatal hernia prior to cardiac surgery must be considered. In the case reported herein, the patient recovered well, but emergent hiatal hernia repair remains high risk. Careful follow-up is also needed for the management of pleural effusions in patients with a chronically indwelling catheter system.

## Abbreviation

PO: postoperative
